# Increased Performances of the Biological Diagnosis of the Antiphospholipid Syndrome by the Use of a Multiplex Assay

**DOI:** 10.1155/2015/983094

**Published:** 2015-05-06

**Authors:** M. Sénant, H. Rostane, F. Fernani-Oukil, F. Hosking, F. Bellery, A. Courchinoux, E. Tartour, L. Darnige, M-A. Dragon-Durey

**Affiliations:** ^1^Laboratoire d'Immunologie, Hôpital Européen Georges Pompidou, Assistance Publique-Hôpitaux de Paris, 20 rue Leblanc, 75908 Paris Cedex 15, France; ^2^Faculté de Médecine, Université Paris Descartes, Paris, France; ^3^Laboratoire d'Hématologie, Hôpital Européen Georges Pompidou, Assistance Publique-Hôpitaux de Paris, 20 rue Leblanc, 75908 Paris Cedex 15, France

## Abstract

Antiphospholipid syndrome (APS) is characterized by development of venous and/or arterial thrombosis and pregnancy morbidity. Biological criteria are the persistent presence of lupus anticoagulant (LA) and/or anti-cardiolipin (aCL) and/or anti-B2GP1 autoantibodies' positivity. The assays' performances are of crucial importance. We evaluated a multiplex assay allowing simultaneous detection of IgG anti-cardiolipin, anti-B2GP1, and anti-factor II. 300 samples were tested. Patients were categorized according to clinical scores of APS from 0 to 3 based on presence or not of arterial or venous thrombosis, fetal loss, and autoimmunity. We used a multiplex assay for APS for simultaneous detection of aCL, anti-B2GP1, and factor II and compared its performances to ELISA assays. Presence of LA was also assessed. We performed a correlation study of the tested assays and compared their clinical efficacy by ROC curve analysis. We obtained significantly higher performances with the multiplex assay than ELISA with higher area under the curve (AUC). The disease rate increased with the number of positive markers from 9% for 1 marker to 100% for 4 markers positive for patients with high risk scores. The multiplex APS assay exhibited higher performances particularly in case of primary APS and is useful for rapid diagnosis of APS.

## 1. Introduction

Antiphospholipid syndrome is a systemic autoimmune disorder clinically characterized by the presence of history of recurrent arterial and/or venous thrombosis and/or pregnancy morbidity such as recurrent miscarriages and fetal losses. The syndrome may affect simultaneously several organs (central nervous system, kidneys, lungs, skin, liver, etc.), defining the life threatening disease “catastrophic antiphospholipid syndrome” (CAPS) [1]. It can be associated with other autoimmune diseases such as SLE but in more than 50% it may occur in isolation [2]. Because of its variable clinical presentation and its devastating consequences, early and rapid diagnosis is crucial. The biological diagnosis is defined by presence of circulating antiphospholipid antibodies. These autoantibodies are directed against anionic phospholipids (cardiolipin, CL) or against protein-phospholipid complexes which can prolong phospholipids-dependent coagulation assays defining the presence of lupus anticoagulant (LA). This is the case of the antibodies directed against the beta 2 glycoprotein 1 (B2GP1).

However, the formal biological diagnosis of this syndrome remains difficult to assert. First, these autoantibodies may be developed transiently during several pathophysiological conditions mainly infections or inflammatory diseases. That is why the international recommendations indicate that the antiphospholipids positivity must be persistent, at least in two samples collected at 12-week interval [3]. But in some severe situation needing rapid diagnosis, these criteria cannot be suitable. Secondly, some patients develop only some kind of autoantibodies mainly isolated aCL or anti-B2GP1 antibodies. This situation leads to the development of research works aiming to improve the diagnosis by studying the epitope mapping [4] or the affinities [5] of the autoantibodies to better identify the real pathogenic ones. Other approaches characterized autoantibodies directed against other phospholipid targets such as the phosphatidylethanolamine (PE) [6] or phosphatidylserine (PS) [7, 8]. The factor II (or prothrombin) has been studied as a candidate autoantigen in APS with controversial results concerning its potential utility for the diagnosis [9, 10].

Another possibility to increase the accuracy of the biological diagnosis of APS is to search for several autoantibodies in the same sample.

This study evaluates a multiplex assay allowing simultaneous detection of IgG anti-cardiolipin, anti-B2GP1, and anti-factor II. We analyzed the performances of this assay and studied the impact of the number of positive autoantibodies on the diagnosis of APS. This work shows that the multiplex FIDIS APS assay exhibits higher performances particularly in case of primary APS and demonstrates that the simultaneous detection of the 3 types of autoantibodies is useful for rapid diagnosis of APS because the presence of 2 or 3 positive markers increases significantly the odds ratio for the clinical presence of APS.

## 2. Material and Methods

### 2.1. Patients

Unselected 300 serum samples were addressed between June and November 2008 to the Laboratory of Immunology of the Hospital Georges Pompidou in Paris, a reference centre for vascular diseases, for routinely detection of anti-cardiolipin (CL) and anti-B2GP1 autoantibody. They were prospectively and simultaneously tested by the routinely used ELISA methods and by a multiplex method detecting IgG against CL, B2GP1, and factor II. The CL antigens were, respectively, purified from bovine (ELISA) and synthetic (multiplex). B2GP1 and prothrombin (factor II) antigens were purified from human plasma. Clinical data from patients and results for LA assays (dRVVT method; dilute Russell's viper venom time, Siemens) were secondarily collected using the software DXCare. Patients were then categorized according to clinical scores of APS from 0 to 3 based on presence or not of arterial or venous thrombosis, pregnancy morbidity, and autoimmunity (Table 1). All symptoms of miscarriage or pregnancy loss, and all thrombotic events, even if isolated, were taken into account. The autoimmune manifestations were mainly SLE (47%), sclerodermia (20%), vasculitis (10%), or other manifestation such as Gougerot-Sjogren syndrome, autoimmune cytopenia, or thyroiditis.

### 2.2. Multiplex Assay

**Figure 1:**
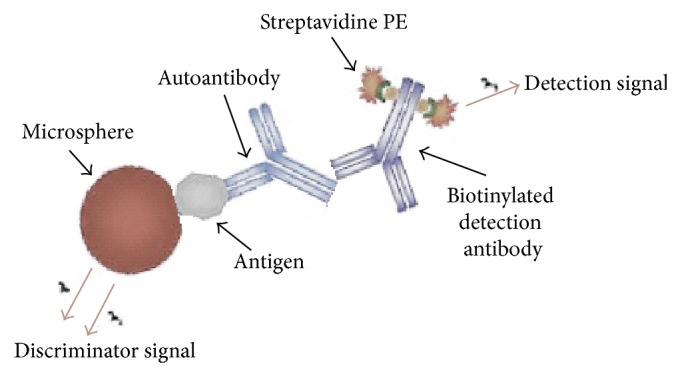
Principle of the multiplex assay. Each of the 3 antigens (cardiolipin, B2GP1, and FII) is coated on specific microspheres defined by a unique spectral signature (discriminator signal). Patients' serums are incubated with a calibrated mixture of the 3 microspheres. The autoantibodies binding is detected by a biotinylated anti-human IgG antibody and a PE labeled streptavidine (detection signal). Both signals are analyzed in a FIDIS flow cytometer.

The multiplex assay tested here was FIDIS APS (Theradiag, Croissy Beaubourg, France) and was performed according to the manufacturer's recommendations. In this assay, each of the 3 tested antigens, that is, cardiolipin, B2GP1, and prothrombin (factor II, FII), is coated on specific microspheres (Luminex) discriminated by a unique spectral signature which will be defined as discriminator signal. Patients' sera were incubated with a calibrated mixture of the 3 types of microspheres in a 96-well microplate. The autoantibodies' binding to the antigens was detected by a biotinylated anti-human IgG antibody and then a PE labeled streptavidine (detection signal). Three washings were performed between each incubation step of one hour at room temperature. After addition of buffer, the plate was read in a FIDIS flow cytometer and both discriminator and detection signals were analyzed using the software MLX-Booster for FIDIS (Figure 1). The cut-off values were >10 UA/mL for anti-B2GP1 and anti-FII. The cut-off value for anti-CL was >10 GPL/mL. The positive thresholds of the FIDIS APS assay were established by 99th percentile of the values obtained by the manufacturer for a normal population (400 samples from blood donors).

### 2.3. Routine ELISA Methods for Detection of IgG against Cardiolipin and B2GP1

The ELISA assay for detection of IgG anti-cardiolipin (Cardiolisa, Theradiag, France) was performed according to the manufacturer's recommendations. In accordance with these, the cut-off values were >10 UGL (99th percentiles).

The detection of IgG anti-B2GP1 was performed by using a home-made ELISA method. Briefly, Nunc MaxiSorp ELISA plates (Nunc, Roskilde, Denmark) were coated with purified human B2GP1 (Stago, France) (10 *μ*g/mL) in PBS overnight at 4°C. After washing and blocking free reactive sites with PBS containing 0,1% tween 20 buffer, the sera to be tested were added at a dilution of 1 : 50 for 1 hour at room temperature. After washing, the plates were incubated for 1 hour at room temperature with a goat anti-human IgG antibody, specific for the *γ* chain, labeled with horseradish peroxidase (Sigma-Aldrich, Steinheim, Germany). After additional washing, enzymatic activity was revealed using the* ortho*-phenylenediamine substrate. Titers of positive samples were expressed as arbitrary units per mL (AU/mL) and calculated using a calibration curve obtained with serial dilutions of a reference positive plasma given an arbitrary titer from 50 to 2 000 AU/mL (optical density ranges from 0.150 to 1.500). The positive threshold was established by the 99th percentile cut-off from 100 individual healthy donors' plasma. This titer was determined to be 120 AU/mL, and titers above this value were considered as positive.

### 2.4. Statistical Analysis

Data were analyzed with MedCalc (version 11.1.1.0) and Graphpad Prism softwares (version 6). A receiver operating characteristic (ROC) curve analysis was performed to compare the methods according to the clinical scores and to find optimal cut-off points. Odds ratios (ORs) with 95% confidence interval (CI) were calculated to compare risk associated to the positivity of one or several autoantibodies.

## 3. Results

### 3.1. Patients

**Figure 2:**
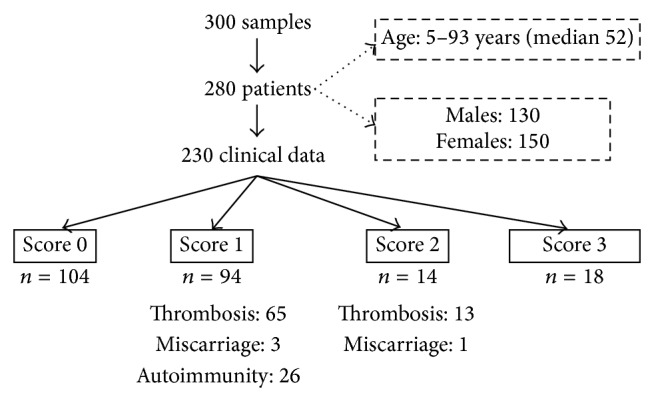
Description of the samples and patients included in the study.

We tested 300 samples from 280 patients. Clinical data were secondarily collected and available for 230 patients (Figure 2). They were aged from 5 to 93 years (median 52) with a sex ratio (M/F) of 0.87. Among them 104 had no history of thrombosis, miscarriage, or fetal loss nor autoimmunity (score 0); 94 had isolated history (score 1) of arterial or venous thrombosis (*n* = 65), miscarriage (*n* = 3), or autoimmunity (*n* = 26); 14 had history of autoimmunity associated with (score 2) thrombosis (*n* = 13) or miscarriage (*n* = 1); finally 18 patients were classified as APS (score 3). Score 4 was attributed to the samples for which no clinical data were available. LA results were available for 207 patients.

### 3.2. Results of the IgG Anti-Cardiolipin, Anti-B2GP1, and Anti-FII by the Multiplex Assay, according to the Clinical Score

**Figure 3:**
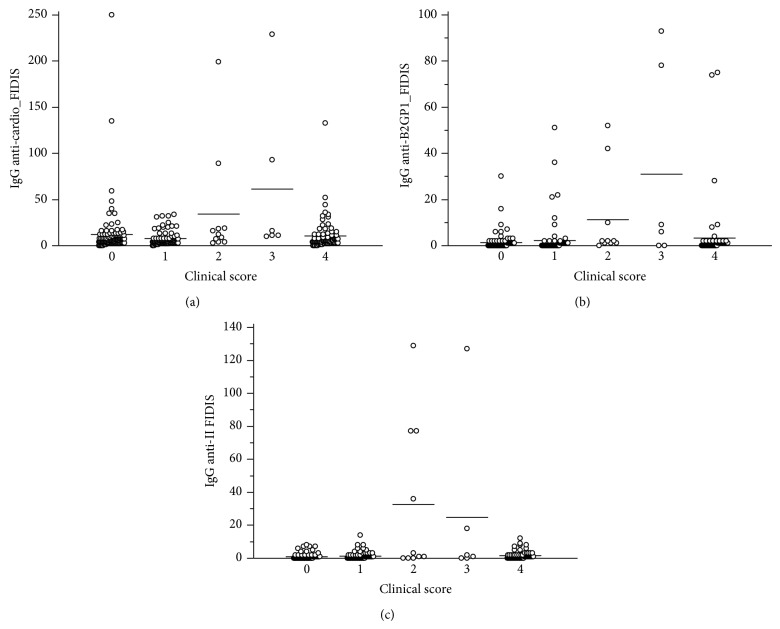
Results of the IgG anti-cardiolipin, anti-B2GP1, and anti-FII according to the APS clinical score.

Presence of IgG anti-cardiolipin was found in 30/102 (29.4%) patients presenting with a clinical score of 0. The median of the titer observed was 13 UGPL (10 to 135 UGPL) in this group. In group 1 patients, we found a positivity in 21/94 (22.3%), with a median titer of 20 UGPL (10–59). Among the patients with a score of 2, 6/14 (42.8%) were positive, with a median titer of 18.5 UGPL (12–250) and all group 3 patients were found positive for IgG anti-cardiolipin (*n* = 18) with a median titer of 31.5 UGPL (10–229) (Figure 3(a)).

IgG anti-B2GP1 were found in none of the 103 patients with a clinical score at 0. Only 4 patients among the 94 (4%) with a score 1 and 3/14 patients from group 2 were positive (median titers: 18.5 and 30, resp.; 10–75 UGPL). In group 3, 13/18 (72%) were positive with a median titer at 42 UGPL (12–289) (Figure 3(b)).

IgG anti-FII were found only in patients from groups 2 (3/14) and 3 (8/18) with a median titer of 77 U/mL (14–659) (Figure 3(c)).

### 3.3. Performances Analysis of the Multiplex versus ELISA Assays

**Figure 4:**
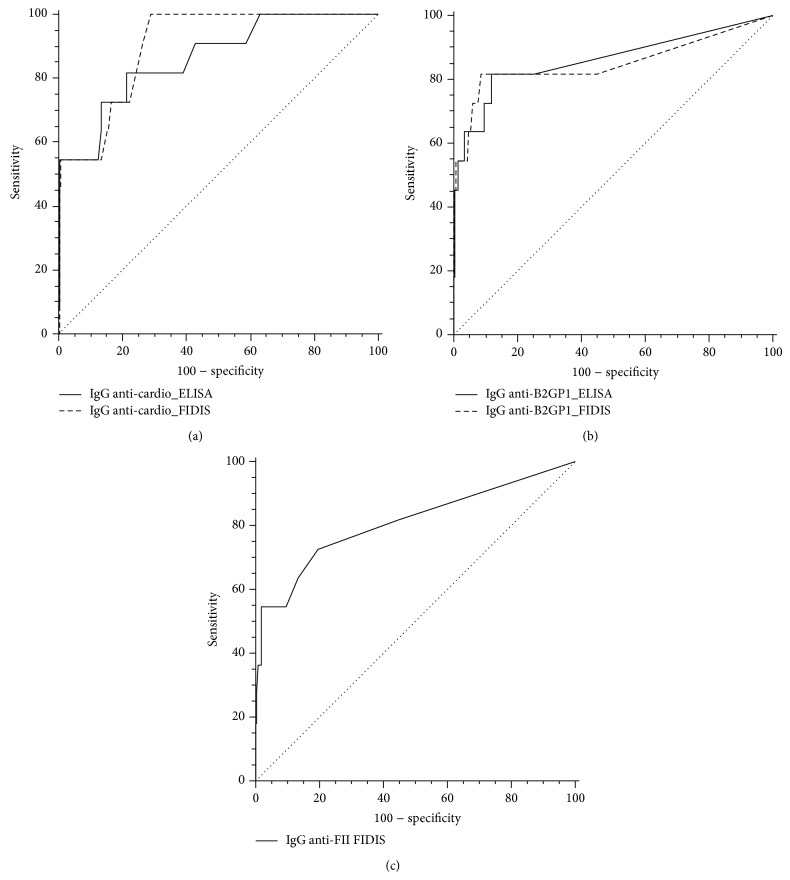
ROC curve analysis with comparison of the ELISA and the FIDIS methods using score 3.

The assays were compared by ROC curve analysis according to the clinical scores described (Figure 4 and Table 2). When we used score 3, only patients with score 3, that is, with confirmed diagnosis of APS, were considered as diseased for the analysis; when we used scores 2/3, patients, with score 2 and patients with score 3, were considered as diseased for the analysis.

For the detection of anti-cardiolipin IgG, we obtained significantly higher performances with the multiplex assay than ELISA when we used score 3 with an area under the curve (AUC) of 0.917 (95% CI 0.873–0.949) versus 0.851 (95% CI 0.798–0.895), with *p* = 0.038. The sensitivity and specificity of the multiplex assay were 100% and 73%, respectively, for a calculated optimal cut-off at 21 UGPL. When we used scores 2/3 for the analysis, the performances of the multiplex and ELISA assays were not different (AUC of 0.795 versus 0.762, resp.).

For the detection of anti-B2GP1 IgG, we observed similar performances for both assays when we used score 3 (AUC of 0.906 versus 0.890, resp.). However when we used scores 2/3, the multiplex assay exhibited higher performances than the ELISA (AUC: 0.863 (95% CI 0.811–0.905) versus 0.715 (95% CI 0.652–0.773), resp.; *p* = 0.001).

For the detection of anti-FII IgG, the AUC was 0.719 (95% CI 0.656–0.776) with score 2/3 and 0.767 (95% CI 0.707–0.820) with score 3. The analysis revealed a sensitivity and a specificity of 50 and 97%, respectively, with an optimal cut-off at 7 U/mL.

### 3.4. Correlation between the Number of Positive Markers and the Clinical Score

We then looked whether the number of positive autoantibodies is correlated with the clinical score.

We calculated first the odds ratio associated with presence of IgG anti-cardiolipin alone or associated with the presence of anti-B2GP1 IgG considering the patients of group 3 or 2/3 as previously described (Tables 3(a) and 3(b)).

When we considered that only patients with score 3 are diseased, the disease rate associated with a positivity of anti-cardiolipin IgG was 24% in the total cohort. The odds ratio associated with this positivity could not be calculated because all patients with score 3 were positive. When we considered that patients with scores 2 and 3 are diseased, the disease rate was 32% and the odds ratio associated with the anti-cardiolipin IgG positivity was 9 (95% IC [3.7–20.5]). The odds ratio increased significantly when the patients were anti-cardiolipin and anti-B2GP1 IgG positive (OR 49, 95% IC [14–162]). This double positivity was associated with an OR of 76 (95% IC [21–273]) when we considered only patients with score 3. The triple positivity (CL, B2GP1, and LA) was associated with an OR of 211 (95% IC [24–1850]) considering the patients with score 3 or 2 and 3 because all patients with triple positivity belonged to group 3 except one who belonged to group 1.

We then studied the influence of the number of positive markers on the diagnosis of APS according to the clinical score.

**Figure 5:**
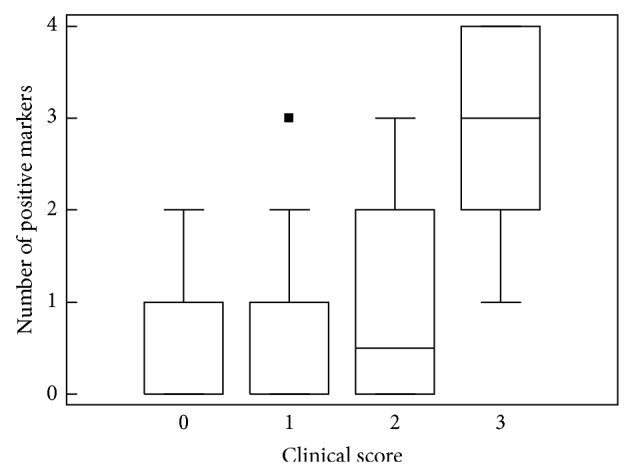
Number of positive markers according to the APS clinical score.

In the group of patients with score 0, no patient exhibited more than one autoantibody except one exhibiting a positivity for CL and LA. In the group with score 1, 4 patients were positive for 2 and one was positive for 3 autoantibodies. In the group with score 3 all patients were positive for at least one antibody with a mean of 2.7 positive markers (Figure 5).

**Figure 6:**
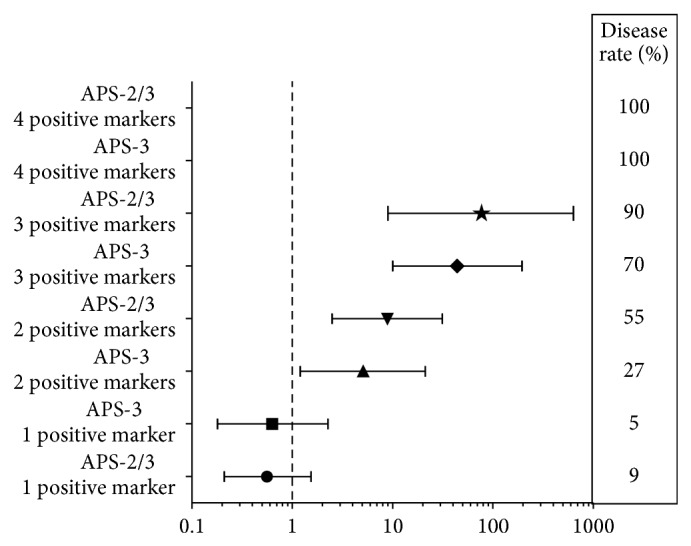
Odds ratio associated with the number of positive markers according to the APS clinical score.

We then calculated the odds ratio associated with the number of positive autoantibodies according to the clinical score (Figure 6). We observed that one isolated positive autoantibody was not associated with a significant risk for the diagnosis of APS whatever the patient groups considered (for group 3, OR = 0.63, 95% IC [0.18–2.27]; for group 2/3 OR = 0.56, 95% IC [0.21–1.54]). The OR increased significantly with the number of positivity. The odds ratios could not be calculated when 4 markers were positive in group 2/3 of patients because all patients were diseased. The same situation was observed for 4 markers with score 3.

## 4. Discussion

Antiphospholipid syndrome is a rare disease that needs strong biological markers because of its severity and its therapeutic management. Indeed, patients suffering from this disease are treated by long term anticoagulation, frequently life-long treatment, with a significant risk of side effects. Even if the international consensus gave us strict classification criteria [3], the biological diagnosis remains difficult for the everyday laboratory work, particularly in case of the first biological evaluation. Indeed, this classification is based on laboratory criteria collected during time (1 positive marker on 2 or more occasions at least 12 weeks apart). The interpretation of the biological results may be difficult because of the absence of any information about the clinical data or because of excessive testing. In our study sampling, which has been prospectively constituted, we failed to collect clinical data for 70 patients (70/230, 30.4%), and 104 tested patients presented neither the clinical diagnosis criteria for APS (no thrombosis, no pregnancy morbidity) nor clinical risk such as autoimmune disease (104/230, 45.2%). This indicates that the APS screening is now considered by the practitioners as a basic testing, leading to the necessity for the biologist to have not only sensitive, but also now specific assays.

Even when one or several clinical criteria are present, the biological markers are frequently insufficient to assert the diagnosis. aCL IgG are known to have a good sensitivity but an important lack of specificity [11]. In our series, they were found not only in, respectively, 22.3% and 42.8% of patients with one or two clinical criteria (scores 1 and 2) but also in 29.4% of patients who presented no clinical criteria (score 0). Furthermore, the titres of aCL failed to be informative because the medians of positive titres observed were not significantly different in the groups: 13, 20, and 18.5 UGLP, in the groups of patients with, respectively, scores 0, 1, and 2. Conversely, the anti-B2GP1 ab positivity shows a lack of sensitivity within our series only 4 and 21% of positive patients among groups 1 and 2, respectively. However none of the patients with score 0 presented with anti-B2GP1 ab, supporting the strong specificity of this marker.

In this study we asked whether the association of several biological markers could help the biological diagnosis. We thus test a multiplex assay allowing the simultaneous detection of IgG directed against CL, B2GP1, and prothrombin, the coagulation factor II. We did not search for autoantibodies of IgM and IgA isotypes because of their lack of specificities [12]. Furthermore we tested presence of IgM in one group of unselected patients (*n* = 147) from our hospital which is specialized in vascular diseases and not in obstetrical morbidity and did not found any sample with isolated IgM autoantibody (data not shown) as found in previous studies [11, 13, 14].

We compared the performances of the assay for the detection of each antigen to the routinely used ELISA tests and we observed comparable or better performances of the multiplex assay as compared to the ELISA assays for the detection of aCL and B2GP1 IgG.

In this assay, the unanimously recognized antigens for APS, cardiolipin, and B2GP1 have been associated with the more controversial prothrombin or factor II (FII). Different studies have been performed on anti-FII antibodies with discordant results according to the studied group. Some studies have been performed in cohorts of patients with primary APS and found high specificity (99.5%) but low sensitivity (19.6%), with an association with thrombosis (OR 69.2) [10], or association with pregnancy loss in a group of women presenting with APS [15]. Another study was performed in a cohort of patients presenting with lupus anticoagulant (LA) and found no association with clinical features of APS and suggested rather a negative predictive value in patients with no IgG directed against both FII and B2GP1 [9]. Among our patients having a clinical score of 3 and LA (*n* = 11), 6 were positive for anti-FII and 5 negative. Other studies were performed in cohorts of patients presenting with SLE and found an association with arterial thrombosis [16, 17]. Overall, in our cohort of unselected patients we found IgG anti-FII only in patients with score 2 or 3 leading to a high specificity (97%) but a low sensitivity (50%) of this marker. Recently, the use of antibodies against phosphatidylserine-dependent antiprothrombin (aPS/PT) has been evaluated by task force scientists (14th International Congress on Antiphospholipid Antibodies, APLA) and it has been concluded that this marker could have a potential importance in routine analysis but harmonization and standardization of these tests remain needed [18].

Thus, the association of this marker with cardiolipin (very sensitive but with low specificity) and B2GP1 (with moderate sensitivity but high specificity) should increase the performance of APS diagnosis. We studied the odd ratio associated with multiple positivity and showed that the simultaneous detection of the 3 types of autoantibodies is useful for rapid diagnosis of APS because presence of 2 or 3 positive markers increases significantly the odds ratio for the clinical presence of APS. The number of positive autoantibodies from 1 to 3 increased the disease rate from 5 to 88%, considering only patients from group 3 as diseased, and from 13 to 100% when we considered patients from group 2 and from group 3 as diseased. When we associated the presence of LA, we observed an increase of the disease rate to 100% whatever the score considered. The association of aCL and anti-B2GP1 positivity increased the odds ratio from 9 to 49 (considering patients from group 2 and from group 3 as diseased). The triple association aCL, anti-B2Gp1, and LA was associated with an odds ratio of 211 in patients with score 3 or score 2/3. However in group 2/3 the disease rate associated with LA positivity alone was 65% as compared to 80% for the double positivity aCL and anti-B2GP1. Only few studies analyzed the impact of the multipositivity of autoantibodies on the performances of the diagnosis. When studying a group of APS patients and a group of healthy blood donors, Hoxha et al. found an increase of the specificity of the biological diagnosis to 100% when they analyzed the simultaneous positivity of aCL, anti-B2GP1, anti-FII, and LA, but with a decreased sensitivity (14.6%), this analysis comprises the IgM and IgG isotypes [10]. In their study, Otomo et al. used the combination of the results of 5 clotting assays and 6 ELISA tests for the detection of antibodies against CL, B2GP1, and phosphatidylserine-dependent antiprothrombin (aPS/PT) of IgM and IgG isotypes to calculate an APS score for thrombosis [19]. However the calculation of this score was quite difficult. They gave a specific score to each positive marker according to the OR associated with its positivity and calculated the total of each score for one patient. With this method, they proposed to help the diagnosis of APS by giving a quantitative and predictive marker of thrombosis. This approach seems very useful for research studies but difficult to apply for the everyday work of one laboratory. It needs to evaluate each marker to attribute its own score on a specific study group. Furthermore, the score attributed to the positivity of high titers of aCL, anti-B2GP1, and aPS/PT antibodies was 20 units for each for a discriminative total of 30; thus in this score the main factors influencing the predictive values kept the positivity of the APS associated antibodies.

Another restriction to the use of this kind of approach is the high volume of blood required as well as the technical time required for the realization of all these assays. For this reason the use of a multiplex assay using microbeads coated with different antigens is an approach which is not time consuming and needs a low sample volume.

## 5. Conclusion

In conclusion, our results show that a multiplex assay is useful for the everyday laboratory work and is efficient for APS diagnosis because the presence of 2 or 3 positive markers increases significantly the odds ratio for clinical APS.

## Figures and Tables

**Table 1:** Clinical score used in the study. Patients were classified according to the presence or not of symptoms of autoimmunity, thrombosis or history of miscarriage.

Score	Clinical criteria
0	No thrombosis, no miscarriage, and no autoimmunity
1	Arterial/venous thrombosis or miscarriage or autoimmunity
2	Arterial/venous thrombosis or miscarriage and autoimmunity
3	Confirmed APS
4	No clinical data available

**Table 2:** Performances of the assays according to the clinical scores used.

Assay	Score used	AUC	95% CI	Sensitivity/specificity (%) at optimal cut-off [U]
Cardio FIDIS	2-3	0.795	0.737–0.846	77/73 [8]
	3	0.917^*^	0.873–0.949^*^	100/73 [9]

Cardiolisa	2-3	0.762	0.702–0.816	65/79 [30]
	3	0.851^*^	0.798–0.895^*^	88/69 [21]

B2GP1 FIDIS	2-3	0.863^**^	0.811–0.905^**^	72/86 [1]
	3	0.906	0.861–0.941	89/93 [4]

B2GP1 ELISA	2-3	0.715^**^	0.652–0.773^**^	59/83 [120]
	3	0.890	0.843–0.928	89/87 [165]

Factor II FIDIS	2-3	0.719	0.656–0.776	41/95 [5]
	3	0.767	0.707–0.820	50/97 [7]

Lupus anticoagulant	2-3	0.785	0.672–0.898	63/94
	3	0.731	0.621–0.841	48/99

Significative difference between cardio FIDIS and ELISA (*p* = 0.038) (^*^) and B2GP1 FIDIS and ELISA (*p* = 0.001) (^**^); AUC: area under the curve.

**(a):** 

Studied group	Number of cases (APS/no APS)	Disease rate (%)	Odds ratio	95% CI	*p*
APS-3 aCL+/B2+/LA	(9/1)	90	211	24–1850	*p* < 10^−4^
APS-3 aCL+/B2+	(13/7)	65	76	21–273	*p* < 10^−4^
APS-3 aCL+/LA	(11/4)	73	82	20.8–322	*p* < 10^−4^
APS-3 B2+/LA	(9/1)	90	211	24–1850	*p* < 10^−4^
APS-3 aCL+	(18/57)	24	NA	NA	NA
APS-3 B2+	(13/7)	65	76	21–273	*p* < 10^−4^
APS-3 LA	(11/9)	55	35	11–113	*p* < 10^−4^

**(b):** 

Studied group	Number of cases (APS/no APS)	Disease rate (%)	Odds ratio	95% CI	*p*
APS-2/3 aCL+/B2+/LA	(9/1)	90	211	24–1850	*p* < 10^−4^
APS-2/3 aCL+/B2+	(16/4)	80	49	14–162	p < 10^−4^
APS-2/3 aCL+/LA	(12/3)	80	39	10–150	p < 10^−4^
APS-2/3 B2+/LA	(9/1)	90	77	9–636	p < 10^−4^
APS-2/3 aCL+	(24/51)	32	9	3.7–20.5	p < 10^−4^
APS-2/3 B2+	(16/4)	80	49	14–162	p < 10^−4^
APS-2/3 LA	(13/7)	65	19	6.7–52	p < 10^−4^
